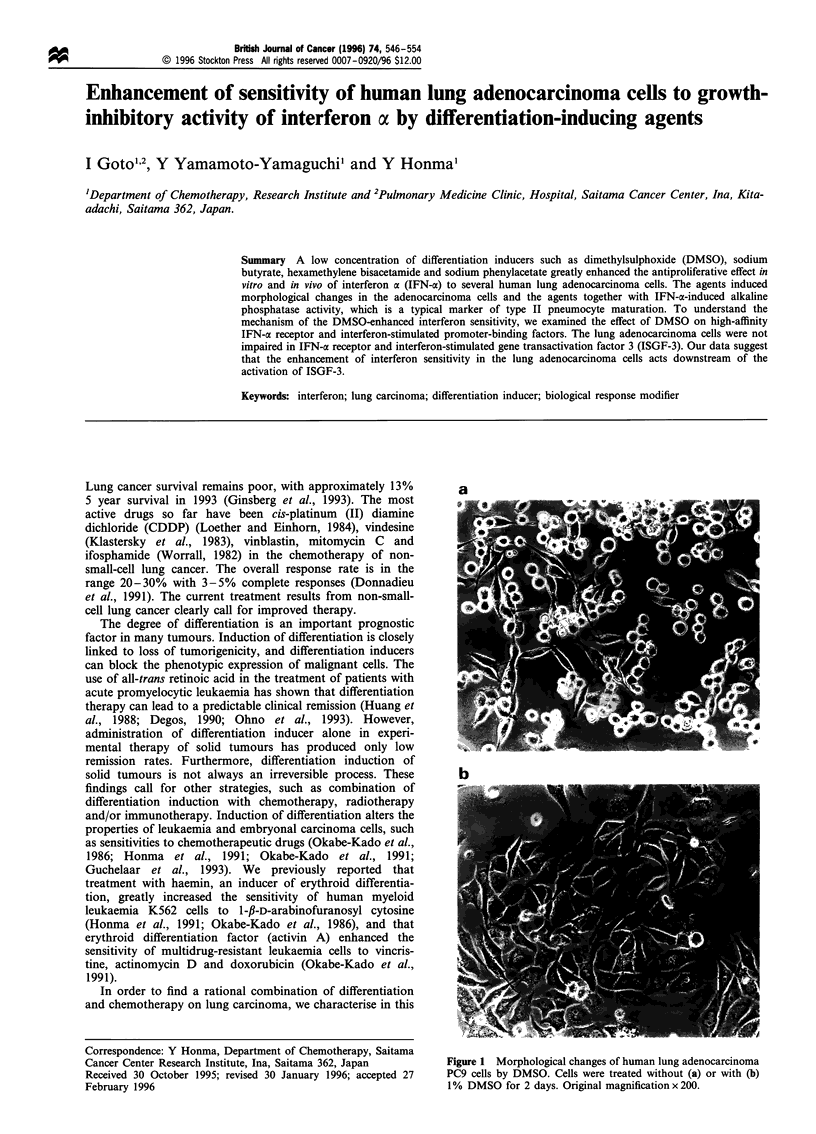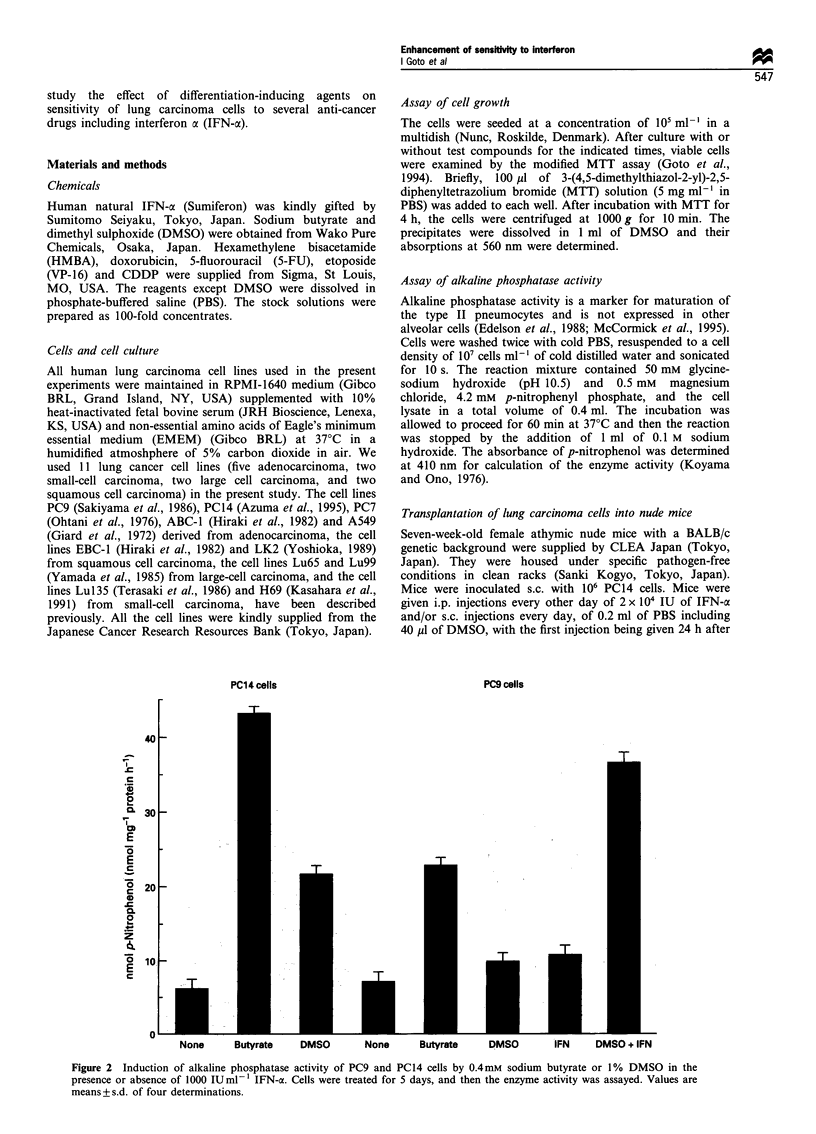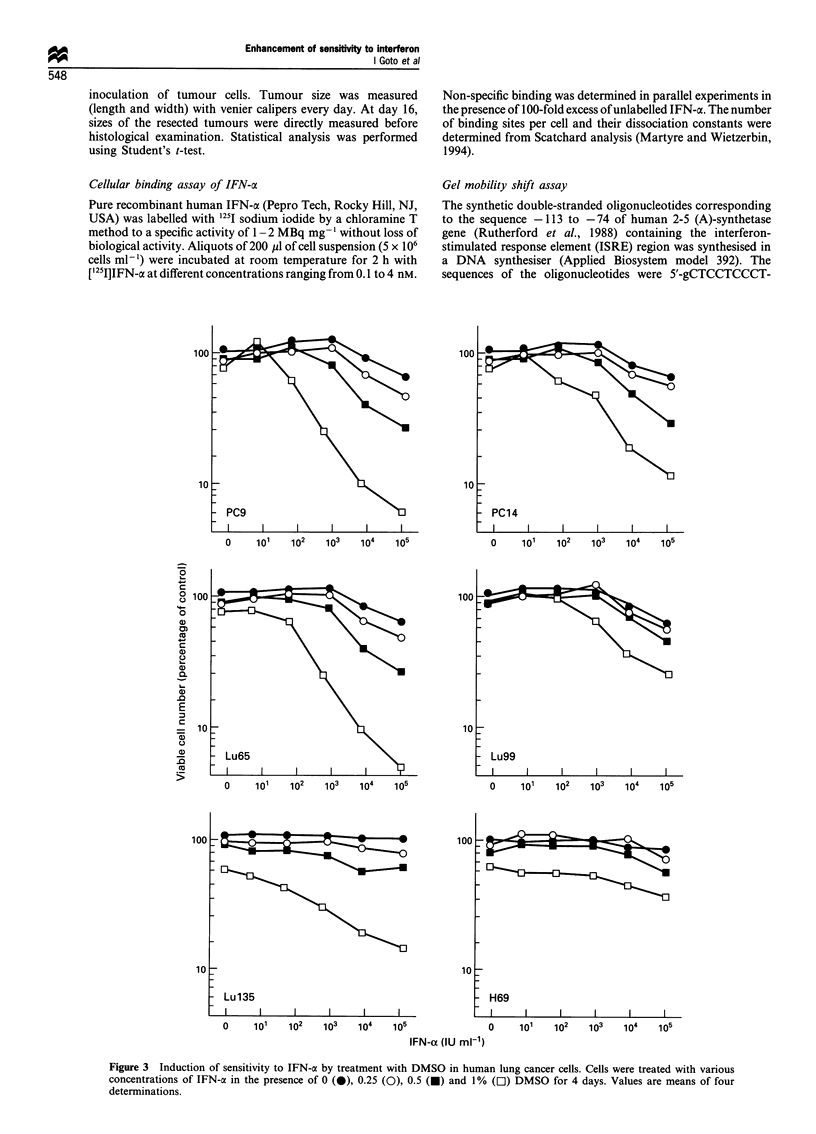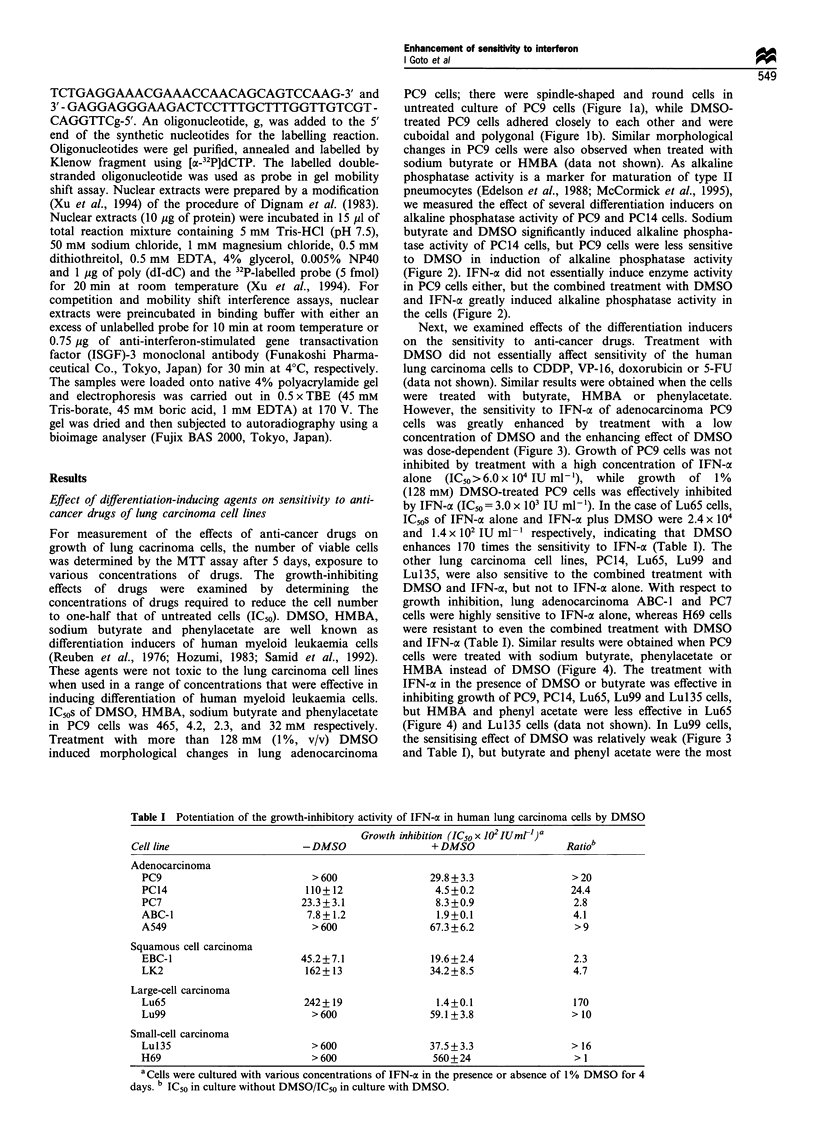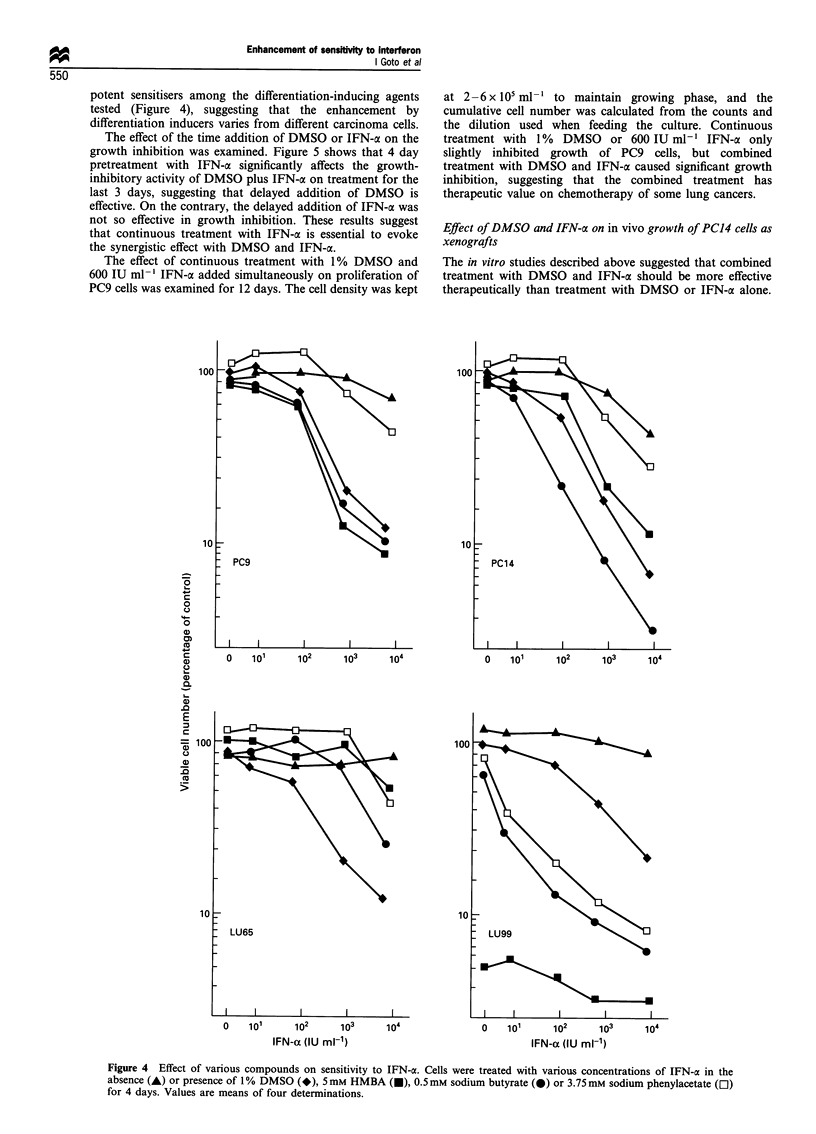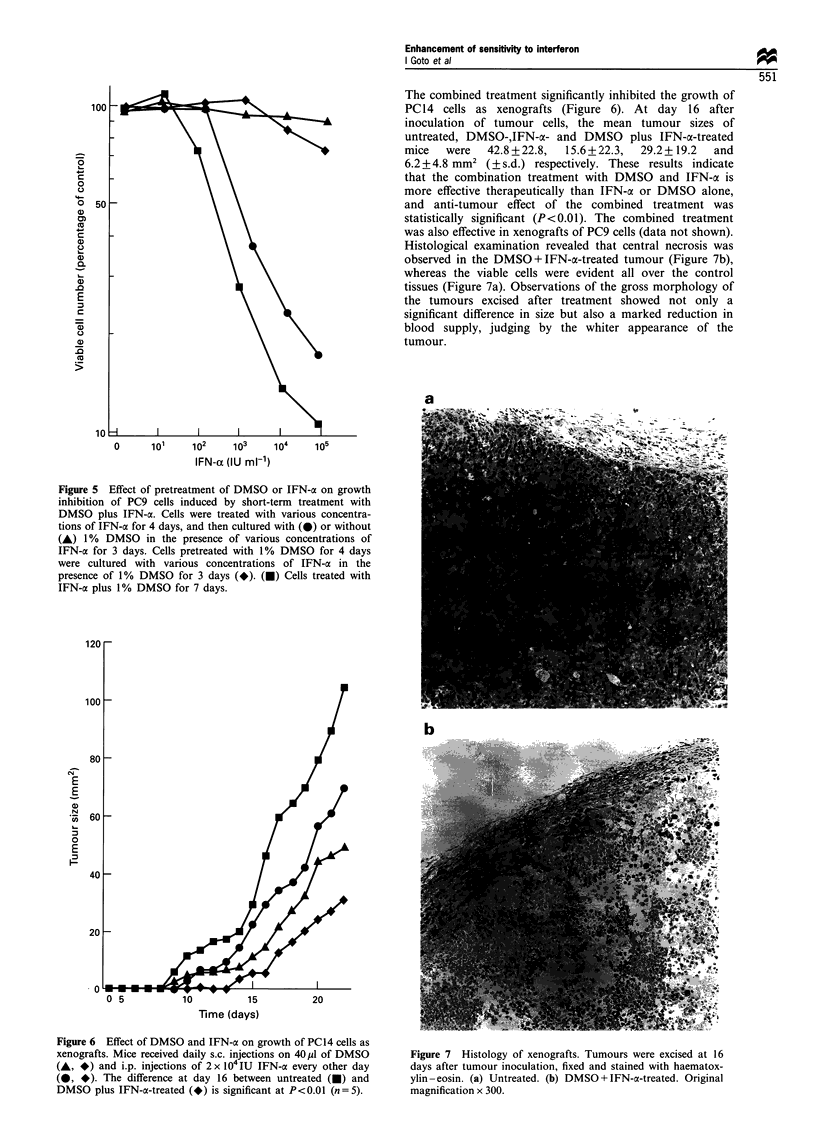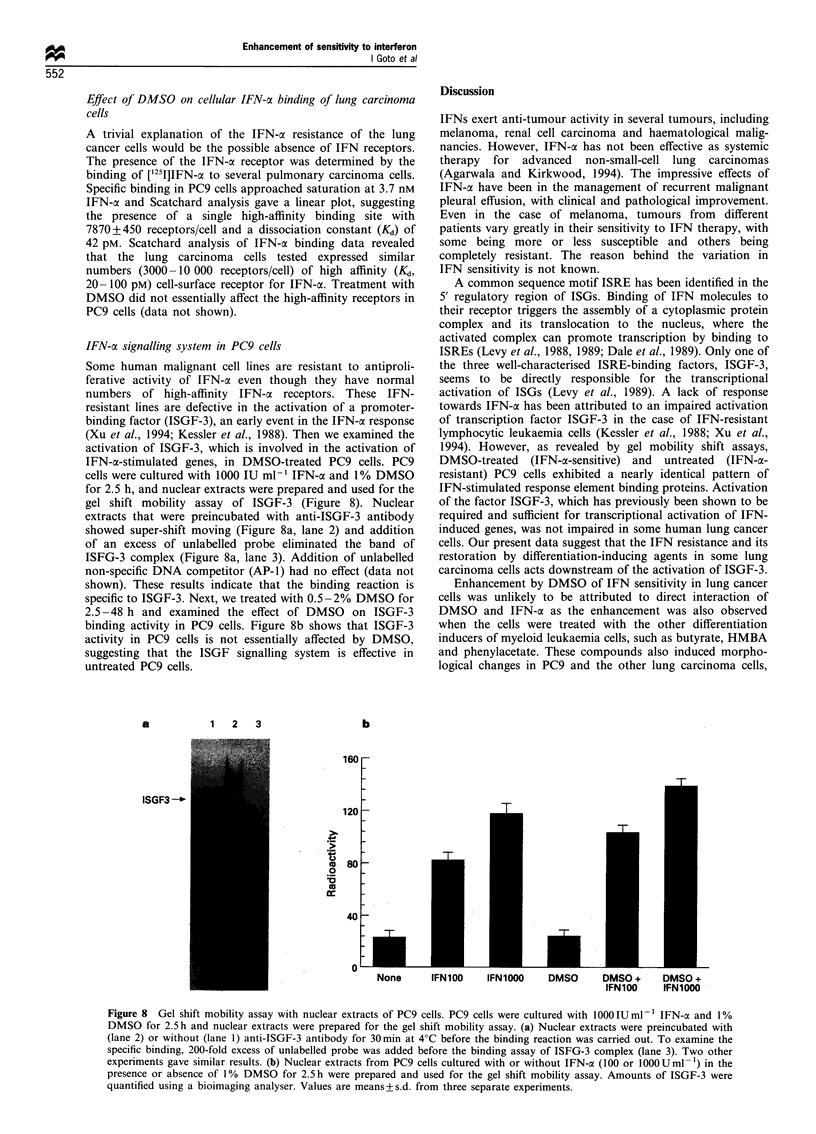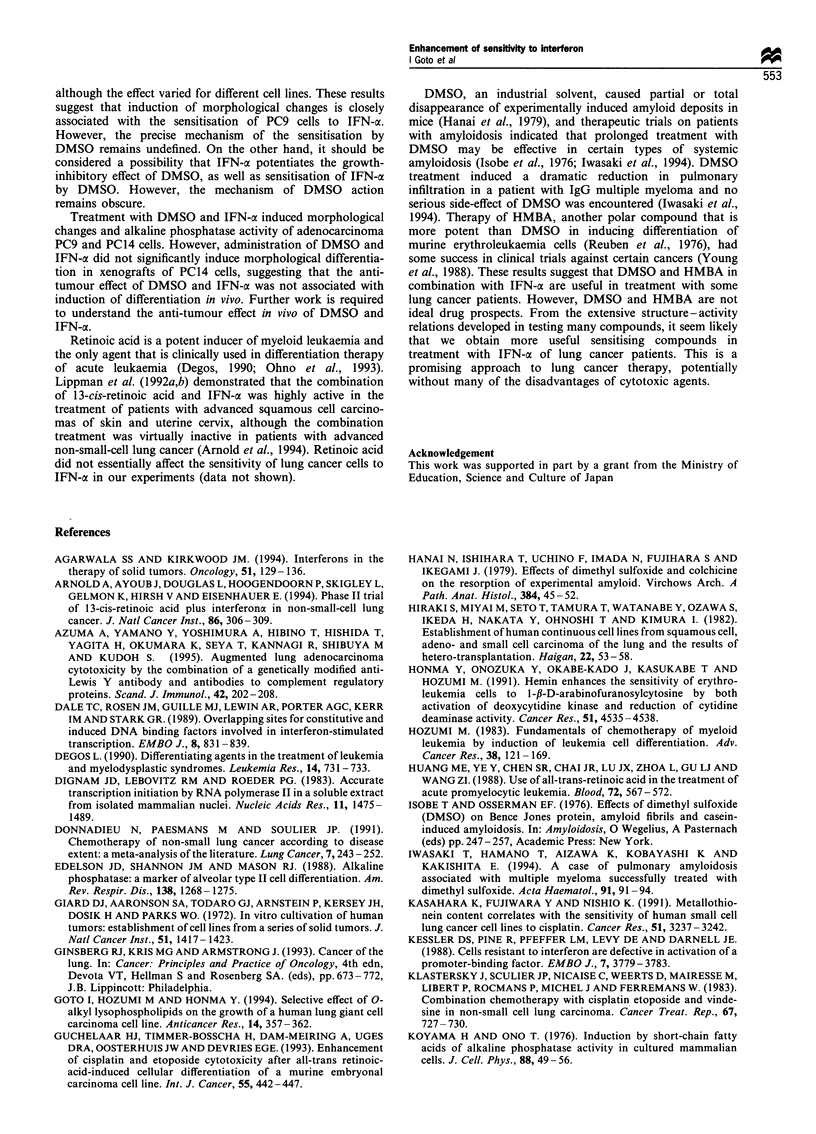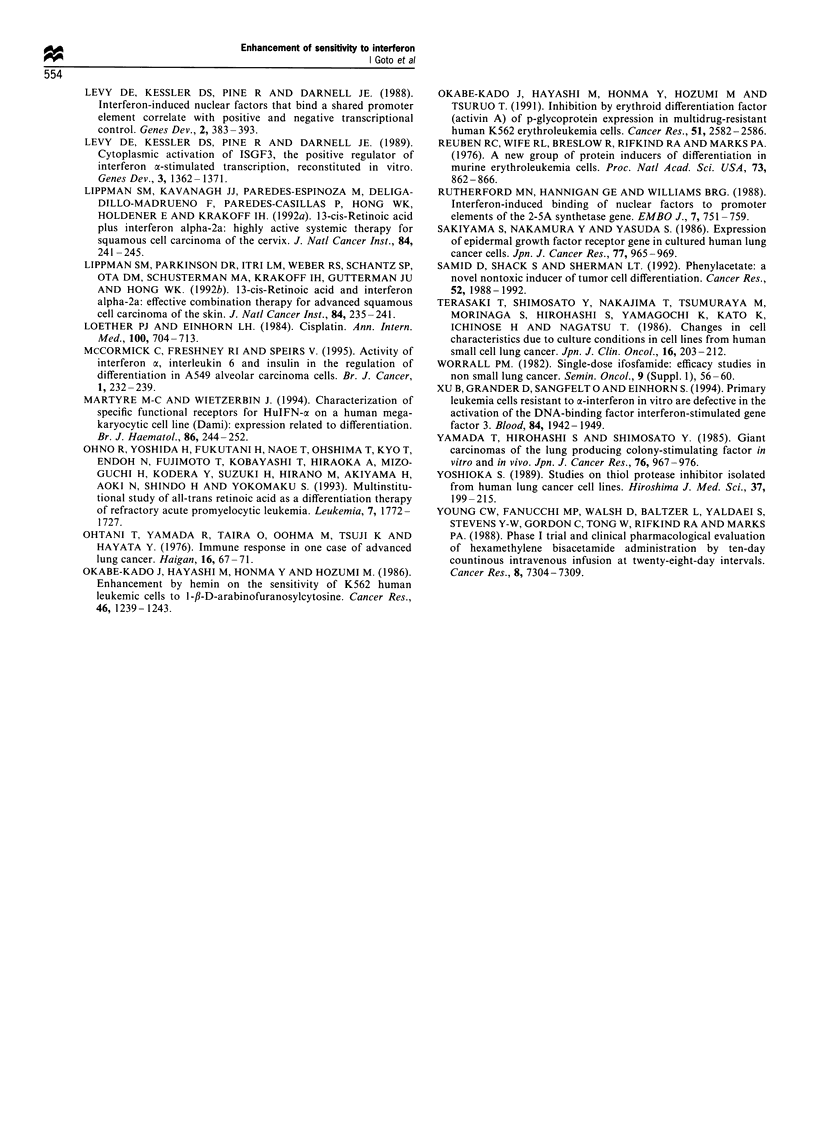# Enhancement of sensitivity of human lung adenocarcinoma cells to growth-inhibitory activity of interferon alpha by differentiation-inducing agents.

**DOI:** 10.1038/bjc.1996.399

**Published:** 1996-08

**Authors:** I. Goto, Y. Yamamoto-Yamaguchi, Y. Honma

**Affiliations:** Department of Chemotherapy, Saitama Cancer Center, Japan.

## Abstract

**Images:**


British Journal of Cancer (1996) 74, 546–554
© 1996 Stockton Press All rights reserved 0007–0920/96 $12.00

### Enhancement of sensitivity of human lung adenocarcinoma cells to growth-inhibitory activity of interferon α by differentiation-inducing agents

I Goto[1,2], Y Yamamoto-Yamaguchi[1] and Y Honma[1]

[1]*Department of Chemotherapy, Research Institute and* [2]*Pulmonary Medicine Clinic, Hospital, Saitama Cancer Center, Ina, Kita-adachi, Saitama 362, Japan.*

**Summary** A low concentration of differentiation inducers such as dimethylsulphoxide (DMSO), sodium butyrate, hexamethylene bisacetamide and sodium phenylacetate greatly enhanced the antiproliferative effect *in vitro* and *in vivo* of interferon α (IFN-α) to several human lung adenocarcinoma cells. The agents induced morphological changes in the adenocarcinoma cells and the agents together with IFN-α-induced alkaline phosphatase activity, which is a typical marker of type II pneumocyte maturation. To understand the mechanism of the DMSO-enhanced interferon sensitivity, we examined the effect of DMSO on high-affinity IFN-α receptor and interferon-stimulated promoter-binding factors. The lung adenocarcinoma cells were not impaired in IFN-α receptor and interferon-stimulated gene transactivation factor 3 (ISGF-3). Our data suggest that the enhancement of interferon sensitivity in the lung adenocarcinoma cells acts downstream of the activation of ISGF-3.

**Keywords:** interferon; lung carcinoma; differentiation inducer; biological response modifier

Lung cancer survival remains poor, with approximately 13% 5 year survival in 1993 (Ginsberg *et al.*, 1993). The most active drugs so far have been *cis*-platinum (II) diamine dichloride (CDDP) (Loether and Einhorn, 1984), vindesine (Klastersky *et al.*, 1983), vinblastin, mitomycin C and ifosphamide (Worrall, 1982) in the chemotherapy of non-small-cell lung cancer. The overall response rate is in the range 20–30% with 3–5% complete responses (Donnadieu *et al.*, 1991). The current treatment results from non-small-cell lung cancer clearly call for improved therapy.

The degree of differentiation is an important prognostic factor in many tumours. Induction of differentiation is closely linked to loss of tumorigenicity, and differentiation inducers can block the phenotypic expression of malignant cells. The use of all-*trans* retinoic acid in the treatment of patients with acute promyelocytic leukaemia has shown that differentiation therapy can lead to a predictable clinical remission (Huang *et al.*, 1988; Degos, 1990; Ohno *et al.*, 1993). However, administration of differentiation inducer alone in experimental therapy of solid tumours has produced only low remission rates. Furthermore, differentiation induction of solid tumours is not always an irreversible process. These findings call for other strategies, such as combination of differentiation induction with chemotherapy, radiotherapy and/or immunotherapy. Induction of differentiation alters the properties of leukaemia and embryonal carcinoma cells, such as sensitivities to chemotherapeutic drugs (Okabe-Kado *et al.*, 1986; Honma *et al.*, 1991; Okabe-Kado *et al.*, 1991; Guchelaar *et al.*, 1993). We previously reported that treatment with haemin, an inducer of erythroid differentiation, greatly increased the sensitivity of human myeloid leukaemia K562 cells to 1-β-D-arabinofuranosyl cytosine (Honma *et al.*, 1991; Okabe-Kado *et al.*, 1986), and that erythroid differentiation factor (activin A) enhanced the sensitivity of multidrug-resistant leukaemia cells to vincristine, actinomycin D and doxorubicin (Okabe-Kado *et al.*, 1991).

In order to find a rational combination of differentiation and chemotherapy on lung carcinoma, we characterise in this

**Figure 1** Morphological changes of human lung adenocarcinoma PC9 cells by DMSO. Cells were treated without (**a**) or with (**b**) 1% DMSO for 2 days. Original magnification × 200.

Correspondence: Y Honma, Department of Chemotherapy, Saitama Cancer Center Research Institute, Ina, Saitama 362, Japan
Received 30 October 1995; revised 30 January 1996; accepted 27 February 1996

study the effect of differentiation-inducing agents on sensitivity of lung carcinoma cells to several anti-cancer drugs including interferon α (IFN-α).

### Materials and methods

### *Chemicals*

Human natural IFN-α (Sumiferon) was kindly gifted by Sumitomo Seiyaku, Tokyo, Japan. Sodium butyrate and dimethyl sulphoxide (DMSO) were obtained from Wako Pure Chemicals, Osaka, Japan. Hexamethylene bisacetamide (HMBA), doxorubicin, 5-fluorouracil (5-FU), etoposide (VP-16) and CDDP were supplied from Sigma, St Louis, MO, USA. The reagents except DMSO were dissolved in phosphate-buffered saline (PBS). The stock solutions were prepared as 100-fold concentrates.

### *Cells and cell culture*

All human lung carcinoma cell lines used in the present experiments were maintained in RPMI-1640 medium (Gibco BRL, Grand Island, NY, USA) supplemented with 10% heat-inactivated fetal bovine serum (JRH Bioscience, Lenexa, KS, USA) and non-essential amino acids of Eagle's minimum essential medium (EMEM) (Gibco BRL) at 37°C in a humidified atmoshphere of 5% carbon dioxide in air. We used 11 lung cancer cell lines (five adenocarcinoma, two small-cell carcinoma, two large cell carcinoma, and two squamous cell carcinoma) in the present study. The cell lines PC9 (Sakiyama *et al.*, 1986), PC14 (Azuma *et al.*, 1995), PC7 (Ohtani *et al.*, 1976), ABC-1 (Hiraki *et al.*, 1982) and A549 (Giard *et al.*, 1972) derived from adenocarcinoma, the cell lines EBC-1 (Hiraki *et al.*, 1982) and LK2 (Yoshioka, 1989) from squamous cell carcinoma, the cell lines Lu65 and Lu99 (Yamada *et al.*, 1985) from large-cell carcinoma, and the cell lines Lu135 (Terasaki *et al.*, 1986) and H69 (Kasahara *et al.*, 1991) from small-cell carcinoma, have been described previously. All the cell lines were kindly supplied from the Japanese Cancer Research Resources Bank (Tokyo, Japan).

### *Assay of cell growth*

The cells were seeded at a concentration of $10^5$ $ml^{-1}$ in a multidish (Nunc, Roskilde, Denmark). After culture with or without test compounds for the indicated times, viable cells were examined by the modified MTT assay (Goto *et al.*, 1994). Briefly, 100 $\mu l$ of 3-(4,5-dimethylthiazol-2-yl)-2,5-diphenyltetrazolium bromide (MTT) solution (5 mg $ml^{-1}$ in PBS) was added to each well. After incubation with MTT for 4 h, the cells were centrifuged at 1000 ***g*** for 10 min. The precipitates were dissolved in 1 ml of DMSO and their absorptions at 560 nm were determined.

### *Assay of alkaline phosphatase activity*

Alkaline phosphatase activity is a marker for maturation of the type II pneumocytes and is not expressed in other alveolar cells (Edelson *et al.*, 1988; McCormick *et al.*, 1995). Cells were washed twice with cold PBS, resuspended to a cell density of $10^7$ cells $ml^{-1}$ of cold distilled water and sonicated for 10 s. The reaction mixture contained 50 mM glycine-sodium hydroxide (pH 10.5) and 0.5 mM magnesium chloride, 4.2 mM *p*-nitrophenyl phosphate, and the cell lysate in a total volume of 0.4 ml. The incubation was allowed to proceed for 60 min at 37°C and then the reaction was stopped by the addition of 1 ml of 0.1 M sodium hydroxide. The absorbance of *p*-nitrophenol was determined at 410 nm for calculation of the enzyme activity (Koyama and Ono, 1976).

### *Transplantation of lung carcinoma cells into nude mice*

Seven-week-old female athymic nude mice with a BALB/c genetic background were supplied by CLEA Japan (Tokyo, Japan). They were housed under specific pathogen-free conditions in clean racks (Sanki Kogyo, Tokyo, Japan). Mice were inoculated s.c. with $10^6$ PC14 cells. Mice were given i.p. injections every other day of $2 \times 10^4$ IU of IFN-α and/or s.c. injections every day, of 0.2 ml of PBS including 40 $\mu l$ of DMSO, with the first injection being given 24 h after

**Figure 2** Induction of alkaline phosphatase activity of PC9 and PC14 cells by 0.4 mM sodium butyrate or 1% DMSO in the presence or absence of 1000 IU $ml^{-1}$ IFN-α. Cells were treated for 5 days, and then the enzyme activity was assayed. Values are means ± s.d. of four determinations.

inoculation of tumour cells. Tumour size was measured (length and width) with venier calipers every day. At day 16, sizes of the resected tumours were directly measured before histological examination. Statistical analysis was performed using Student's $t$-test.

*Cellular binding assay of IFN-α*

Pure recombinant human IFN-α (Pepro Tech, Rocky Hill, NJ, USA) was labelled with $^{125}$I sodium iodide by a chloramine T method to a specific activity of 1–2 MBq mg$^{-1}$ without loss of biological activity. Aliquots of 200 μl of cell suspension (5 × 10$^{6}$ cells ml$^{-1}$) were incubated at room temperature for 2 h with [$^{125}$I]IFN-α at different concentrations ranging from 0.1 to 4 nM. Non-specific binding was determined in parallel experiments in the presence of 100-fold excess of unlabelled IFN-α. The number of binding sites per cell and their dissociation constants were determined from Scatchard analysis (Martyre and Wietzerbin, 1994).

*Gel mobility shift assay*

The synthetic double-stranded oligonucleotides corresponding to the sequence −113 to −74 of human 2-5 (A)-synthetase gene (Rutherford et al., 1988) containing the interferon-stimulated response element (ISRE) region was synthesised in a DNA synthesiser (Applied Biosystem model 392). The sequences of the oligonucleotides were 5′-gCTCCTCCCT-

**Figure 3** Induction of sensitivity to IFN-α by treatment with DMSO in human lung cancer cells. Cells were treated with various concentrations of IFN-α in the presence of 0 (●), 0.25 (○), 0.5 (■) and 1% (□) DMSO for 4 days. Values are means of four determinations.

TCTGAGGAAACGAAACCAACAGCAGTCCAAG-3′ and 3′-GAGGAGGGAAGACTCCTTTGCTTTGGTTGTCGT-CAGGTTCg-5′. An oligonucleotide, g, was added to the 5′ end of the synthetic nucleotides for the labelling reaction. Oligonucleotides were gel purified, annealed and labelled by Klenow fragment using [α-$^{32}$P]dCTP. The labelled double-stranded oligonucleotide was used as probe in gel mobility shift assay. Nuclear extracts were prepared by a modification (Xu *et al.*, 1994) of the procedure of Dignam *et al.* (1983). Nuclear extracts (10 μg of protein) were incubated in 15 μl of total reaction mixture containing 5 mM Tris-HCl (pH 7.5), 50 mM sodium chloride, 1 mM magnesium chloride, 0.5 mM dithiothreitol, 0.5 mM EDTA, 4% glycerol, 0.005% NP40 and 1 μg of poly (dI-dC) and the $^{32}$P-labelled probe (5 fmol) for 20 min at room temperature (Xu *et al.*, 1994). For competition and mobility shift interference assays, nuclear extracts were preincubated in binding buffer with either an excess of unlabelled probe for 10 min at room temperature or 0.75 μg of anti-interferon-stimulated gene transactivation factor (ISGF)-3 monoclonal antibody (Funakoshi Pharmaceutical Co., Tokyo, Japan) for 30 min at 4°C, respectively. The samples were loaded onto native 4% polyacrylamide gel and electrophoresis was carried out in 0.5 × TBE (45 mM Tris-borate, 45 mM boric acid, 1 mM EDTA) at 170 V. The gel was dried and then subjected to autoradiography using a bioimage analyser (Fujix BAS 2000, Tokyo, Japan).

### Results

### *Effect of differentiation-inducing agents on sensitivity to anti-cancer drugs of lung carcinoma cell lines*

For measurement of the effects of anti-cancer drugs on growth of lung cacrinoma cells, the number of viable cells was determined by the MTT assay after 5 days, exposure to various concentrations of drugs. The growth-inhibiting effects of drugs were examined by determining the concentrations of drugs required to reduce the cell number to one-half that of untreated cells ($IC_{50}$). DMSO, HMBA, sodium butyrate and phenylacetate are well known as differentiation inducers of human myeloid leukaemia cells (Reuben *et al.*, 1976; Hozumi, 1983; Samid *et al.*, 1992). These agents were not toxic to the lung carcinoma cell lines when used in a range of concentrations that were effective in inducing differentiation of human myeloid leukaemia cells. $IC_{50}$s of DMSO, HMBA, sodium butyrate and phenylacetate in PC9 cells was 465, 4.2, 2.3, and 32 mM respectively. Treatment with more than 128 mM (1%, v/v) DMSO induced morphological changes in lung adenocarcinoma PC9 cells; there were spindle-shaped and round cells in untreated culture of PC9 cells (Figure 1a), while DMSO-treated PC9 cells adhered closely to each other and were cuboidal and polygonal (Figure 1b). Similar morphological changes in PC9 cells were also observed when treated with sodium butyrate or HMBA (data not shown). As alkaline phosphatase activity is a marker for maturation of type II pneumocytes (Edelson *et al.*, 1988; McCormick *et al.*, 1995), we measured the effect of several differentiation inducers on alkaline phosphatase activity of PC9 and PC14 cells. Sodium butyrate and DMSO significantly induced alkaline phosphatase activity of PC14 cells, but PC9 cells were less sensitive to DMSO in induction of alkaline phosphatase activity (Figure 2). IFN-α did not essentially induce enzyme activity in PC9 cells either, but the combined treatment with DMSO and IFN-α greatly induced alkaline phosphatase activity in the cells (Figure 2).

Next, we examined effects of the differentiation inducers on the sensitivity to anti-cancer drugs. Treatment with DMSO did not essentially affect sensitivity of the human lung carcinoma cells to CDDP, VP-16, doxorubicin or 5-FU (data not shown). Similar results were obtained when the cells were treated with butyrate, HMBA or phenylacetate. However, the sensitivity to IFN-α of adenocarcinoma PC9 cells was greatly enhanced by treatment with a low concentration of DMSO and the enhancing effect of DMSO was dose-dependent (Figure 3). Growth of PC9 cells was not inhibited by treatment with a high concentration of IFN-α alone ($IC_{50} > 6.0 \times 10^4$ IU ml$^{-1}$), while growth of 1% (128 mM) DMSO-treated PC9 cells was effectively inhibited by IFN-α ($IC_{50} = 3.0 \times 10^3$ IU ml$^{-1}$). In the case of Lu65 cells, $IC_{50}$s of IFN-α alone and IFN-α plus DMSO were $2.4 \times 10^4$ and $1.4 \times 10^2$ IU ml$^{-1}$ respectively, indicating that DMSO enhances 170 times the sensitivity to IFN-α (Table I). The other lung carcinoma cell lines, PC14, Lu65, Lu99 and Lu135, were also sensitive to the combined treatment with DMSO and IFN-α, but not to IFN-α alone. With respect to growth inhibition, lung adenocarcinoma ABC-1 and PC7 cells were highly sensitive to IFN-α alone, whereas H69 cells were resistant to even the combined treatment with DMSO and IFN-α (Table I). Similar results were obtained when PC9 cells were treated with sodium butyrate, phenylacetate or HMBA instead of DMSO (Figure 4). The treatment with IFN-α in the presence of DMSO or butyrate was effective in inhibiting growth of PC9, PC14, Lu65, Lu99 and Lu135 cells, but HMBA and phenyl acetate were less effective in Lu65 (Figure 4) and Lu135 cells (data not shown). In Lu99 cells, the sensitising effect of DMSO was relatively weak (Figure 3 and Table I), but butyrate and phenyl acetate were the most

**Table I** Potentiation of the growth-inhibitory activity of IFN-α in human lung carcinoma cells by DMSO

| | Growth inhibition ($IC_{50} \times 10^2$ IU ml$^{-1}$)[a] | | |
|---|---|---|---|
| *Cell line* | *−DMSO* | *+DMSO* | *Ratio*[b] |
| Adenocarcinoma | | | |
| PC9 | >600 | 29.8±3.3 | >20 |
| PC14 | 110±12 | 4.5±0.2 | 24.4 |
| PC7 | 23.3±3.1 | 8.3±0.9 | 2.8 |
| ABC-1 | 7.8±1.2 | 1.9±0.1 | 4.1 |
| A549 | >600 | 67.3±6.2 | >9 |
| Squamous cell carcinoma | | | |
| EBC-1 | 45.2±7.1 | 19.6±2.4 | 2.3 |
| LK2 | 162±13 | 34.2±8.5 | 4.7 |
| Large-cell carcinoma | | | |
| Lu65 | 242±19 | 1.4±0.1 | 170 |
| Lu99 | >600 | 59.1±3.8 | >10 |
| Small-cell carcinoma | | | |
| Lu135 | >600 | 37.5±3.3 | >16 |
| H69 | >600 | 560±24 | >1 |

[a] Cells were cultured with various concentrations of IFN-α in the presence or absence of 1% DMSO for 4 days. [b] $IC_{50}$ in culture without DMSO/$IC_{50}$ in culture with DMSO.

potent sensitisers among the differentiation-inducing agents tested (Figure 4), suggesting that the enhancement by differentiation inducers varies from different carcinoma cells.

The effect of the time addition of DMSO or IFN-α on the growth inhibition was examined. Figure 5 shows that 4 day pretreatment with IFN-α significantly affects the growth-inhibitory activity of DMSO plus IFN-α on treatment for the last 3 days, suggesting that delayed addition of DMSO is effective. On the contrary, the delayed addition of IFN-α was not so effective in growth inhibition. These results suggest that continuous treatment with IFN-α is essential to evoke the synergistic effect with DMSO and IFN-α.

The effect of continuous treatment with 1% DMSO and 600 IU $ml^{-1}$ IFN-α added simultaneously on proliferation of PC9 cells was examined for 12 days. The cell density was kept at $2-6 \times 10^5$ $ml^{-1}$ to maintain growing phase, and the cumulative cell number was calculated from the counts and the dilution used when feeding the culture. Continuous treatment with 1% DMSO or 600 IU $ml^{-1}$ IFN-α only slightly inhibited growth of PC9 cells, but combined treatment with DMSO and IFN-α caused significant growth inhibition, suggesting that the combined treatment has therapeutic value on chemotherapy of some lung cancers.

### *Effect of DMSO and IFN-α on* in vivo *growth of PC14 cells as xenografts*

The *in vitro* studies described above suggested that combined treatment with DMSO and IFN-α should be more effective therapeutically than treatment with DMSO or IFN-α alone.

**Figure 4** Effect of various compounds on sensitivity to IFN-α. Cells were treated with various concentrations of IFN-α in the absence (▲) or presence of 1% DMSO (◆), 5 mM HMBA (■), 0.5 mM sodium butyrate (●) or 3.75 mM sodium phenylacetate (□) for 4 days. Values are means of four determinations.

**Figure 5** Effect of pretreatment of DMSO or IFN-α on growth inhibition of PC9 cells induced by short-term treatment with DMSO plus IFN-α. Cells were treated with various concentrations of IFN-α for 4 days, and then cultured with (●) or without (▲) 1% DMSO in the presence of various concentrations of IFN-α for 3 days. Cells pretreated with 1% DMSO for 4 days were cultured with various concentrations of IFN-α in the presence of 1% DMSO for 3 days (◆). (■) Cells treated with IFN-α plus 1% DMSO for 7 days.

**Figure 6** Effect of DMSO and IFN-α on growth of PC14 cells as xenografts. Mice received daily s.c. injections on 40 $\mu$l of DMSO (▲, ◆) and i.p. injections of $2 \times 10^4$ IU IFN-α every other day (●, ◆). The difference at day 16 between untreated (■) and DMSO plus IFN-α-treated (◆) is significant at $P<0.01$ ($n=5$).

The combined treatment significantly inhibited the growth of PC14 cells as xenografts (Figure 6). At day 16 after inoculation of tumour cells, the mean tumour sizes of untreated, DMSO-,IFN-α- and DMSO plus IFN-α-treated mice were $42.8 \pm 22.8$, $15.6 \pm 22.3$, $29.2 \pm 19.2$ and $6.2 \pm 4.8$ mm² ($\pm$s.d.) respectively. These results indicate that the combination treatment with DMSO and IFN-α is more effective therapeutically than IFN-α or DMSO alone, and anti-tumour effect of the combined treatment was statistically significant ($P<0.01$). The combined treatment was also effective in xenografts of PC9 cells (data not shown). Histological examination revealed that central necrosis was observed in the DMSO + IFN-α-treated tumour (Figure 7b), whereas the viable cells were evident all over the control tissues (Figure 7a). Observations of the gross morphology of the tumours excised after treatment showed not only a significant difference in size but also a marked reduction in blood supply, judging by the whiter appearance of the tumour.

**Figure 7** Histology of xenografts. Tumours were excised at 16 days after tumour inoculation, fixed and stained with haematoxylin–eosin. (**a**) Untreated. (**b**) DMSO + IFN-α-treated. Original magnification × 300.

### *Effect of DMSO on cellular IFN-α binding of lung carcinoma cells*

A trivial explanation of the IFN-α resistance of the lung cancer cells would be the possible absence of IFN receptors. The presence of the IFN-α receptor was determined by the binding of [$^{125}$I]IFN-α to several pulmonary carcinoma cells. Specific binding in PC9 cells approached saturation at 3.7 nM IFN-α and Scatchard analysis gave a linear plot, suggesting the presence of a single high-affinity binding site with $7870 \pm 450$ receptors/cell and a dissociation constant ($K_d$) of 42 pM. Scatchard analysis of IFN-α binding data revealed that the lung carcinoma cells tested expressed similar numbers (3000–10 000 receptors/cell) of high affinity ($K_d$, 20–100 pM) cell-surface receptor for IFN-α. Treatment with DMSO did not essentially affect the high-affinity receptors in PC9 cells (data not shown).

### *IFN-α signalling system in PC9 cells*

Some human malignant cell lines are resistant to antiproliferative activity of IFN-α even though they have normal numbers of high-affinity IFN-α receptors. These IFN-resistant lines are defective in the activation of a promoter-binding factor (ISGF-3), an early event in the IFN-α response (Xu *et al.*, 1994; Kessler *et al.*, 1988). Then we examined the activation of ISGF-3, which is involved in the activation of IFN-α-stimulated genes, in DMSO-treated PC9 cells. PC9 cells were cultured with 1000 IU ml$^{-1}$ IFN-α and 1% DMSO for 2.5 h, and nuclear extracts were prepared and used for the gel shift mobility assay of ISGF-3 (Figure 8). Nuclear extracts that were preincubated with anti-ISGF-3 antibody showed super-shift moving (Figure 8a, lane 2) and addition of an excess of unlabelled probe eliminated the band of ISFG-3 complex (Figure 8a, lane 3). Addition of unlabelled non-specific DNA competitor (AP-1) had no effect (data not shown). These results indicate that the binding reaction is specific to ISGF-3. Next, we treated with 0.5–2% DMSO for 2.5–48 h and examined the effect of DMSO on ISGF-3 binding activity in PC9 cells. Figure 8b shows that ISGF-3 activity in PC9 cells is not essentially affected by DMSO, suggesting that the ISGF signalling system is effective in untreated PC9 cells.

### Discussion

IFNs exert anti-tumour activity in several tumours, including melanoma, renal cell carcinoma and haematological malignancies. However, IFN-α has not been effective as systemic therapy for advanced non-small-cell lung carcinomas (Agarwala and Kirkwood, 1994). The impressive effects of IFN-α have been in the management of recurrent malignant pleural effusion, with clinical and pathological improvement. Even in the case of melanoma, tumours from different patients vary greatly in their sensitivity to IFN therapy, with some being more or less susceptible and others being completely resistant. The reason behind the variation in IFN sensitivity is not known.

A common sequence motif ISRE has been identified in the 5′ regulatory region of ISGs. Binding of IFN molecules to their receptor triggers the assembly of a cytoplasmic protein complex and its translocation to the nucleus, where the activated complex can promote transcription by binding to ISREs (Levy *et al.*, 1988, 1989; Dale *et al.*, 1989). Only one of the three well-characterised ISRE-binding factors, ISGF-3, seems to be directly responsible for the transcriptional activation of ISGs (Levy *et al.*, 1989). A lack of response towards IFN-α has been attributed to an impaired activation of transcription factor ISGF-3 in the case of IFN-resistant lymphocytic leukaemia cells (Kessler *et al.*, 1988; Xu *et al.*, 1994). However, as revealed by gel mobility shift assays, DMSO-treated (IFN-α-sensitive) and untreated (IFN-α-resistant) PC9 cells exhibited a nearly identical pattern of IFN-stimulated response element binding proteins. Activation of the factor ISGF-3, which has previously been shown to be required and sufficient for transcriptional activation of IFN-induced genes, was not impaired in some human lung cancer cells. Our present data suggest that the IFN resistance and its restoration by differentiation-inducing agents in some lung carcinoma cells acts downstream of the activation of ISGF-3.

Enhancement by DMSO of IFN sensitivity in lung cancer cells was unlikely to be attributed to direct interaction of DMSO and IFN-α as the enhancement was also observed when the cells were treated with the other differentiation inducers of myeloid leukaemia cells, such as butyrate, HMBA and phenylacetate. These compounds also induced morphological changes in PC9 and the other lung carcinoma cells,

**Figure 8** Gel shift mobility assay with nuclear extracts of PC9 cells. PC9 cells were cultured with 1000 IU ml$^{-1}$ IFN-α and 1% DMSO for 2.5 h and nuclear extracts were prepared for the gel shift mobility assay. (**a**) Nuclear extracts were preincubated with (lane 2) or without (lane 1) anti-ISGF-3 antibody for 30 min at 4°C before the binding reaction was carried out. To examine the specific binding, 200-fold excess of unlabelled probe was added before the binding assay of ISFG-3 complex (lane 3). Two other experiments gave similar results. (**b**) Nuclear extracts from PC9 cells cultured with or without IFN-α (100 or 1000 U ml$^{-1}$) in the presence or absence of 1% DMSO for 2.5 h were prepared and used for the gel shift mobility assay. Amounts of ISGF-3 were quantified using a bioimaging analyser. Values are means ± s.d. from three separate experiments.

although the effect varied for different cell lines. These results suggest that induction of morphological changes is closely associated with the sensitisation of PC9 cells to IFN-α. However, the precise mechanism of the sensitisation by DMSO remains undefined. On the other hand, it should be considered a possibility that IFN-α potentiates the growth-inhibitory effect of DMSO, as well as sensitisation of IFN-α by DMSO. However, the mechanism of DMSO action remains obscure.

Treatment with DMSO and IFN-α induced morphological changes and alkaline phosphatase activity of adenocarcinoma PC9 and PC14 cells. However, administration of DMSO and IFN-α did not significantly induce morphological differentiation in xenografts of PC14 cells, suggesting that the anti-tumour effect of DMSO and IFN-α was not associated with induction of differentiation *in vivo*. Further work is required to understand the anti-tumour effect *in vivo* of DMSO and IFN-α.

Retinoic acid is a potent inducer of myeloid leukaemia and the only agent that is clinically used in differentiation therapy of acute leukaemia (Degos, 1990; Ohno *et al.*, 1993). Lippman *et al.* (1992*a,b*) demonstrated that the combination of 13-*cis*-retinoic acid and IFN-α was highly active in the treatment of patients with advanced squamous cell carcinomas of skin and uterine cervix, although the combination treatment was virtually inactive in patients with advanced non-small-cell lung cancer (Arnold *et al.*, 1994). Retinoic acid did not essentially affect the sensitivity of lung cancer cells to IFN-α in our experiments (data not shown).

DMSO, an industrial solvent, caused partial or total disappearance of experimentally induced amyloid deposits in mice (Hanai *et al.*, 1979), and therapeutic trials on patients with amyloidosis indicated that prolonged treatment with DMSO may be effective in certain types of systemic amyloidosis (Isobe *et al.*, 1976; Iwasaki *et al.*, 1994). DMSO treatment induced a dramatic reduction in pulmonary infiltration in a patient with IgG multiple myeloma and no serious side-effect of DMSO was encountered (Iwasaki *et al.*, 1994). Therapy of HMBA, another polar compound that is more potent than DMSO in inducing differentiation of murine erythroleukaemia cells (Reuben *et al.*, 1976), had some success in clinical trials against certain cancers (Young *et al.*, 1988). These results suggest that DMSO and HMBA in combination with IFN-α are useful in treatment with some lung cancer patients. However, DMSO and HMBA are not ideal drug prospects. From the extensive structure–activity relations developed in testing many compounds, it seem likely that we obtain more useful sensitising compounds in treatment with IFN-α of lung cancer patients. This is a promising approach to lung cancer therapy, potentially without many of the disadvantages of cytotoxic agents.

**Acknowledgement**
This work was supported in part by a grant from the Ministry of Education, Science and Culture of Japan

### References

AGARWALA SS AND KIRKWOOD JM. (1994). Interferons in the therapy of solid tumors. *Oncology*, **51**, 129–136.

ARNOLD A, AYOUB J, DOUGLAS L, HOOGENDOORN P, SKIGLEY L, GELMON K, HIRSH V AND EISENHAUER E. (1994). Phase II trial of 13-cis-retinoic acid plus interferonα in non-small-cell lung cancer. *J. Natl Cancer Inst.*, **86**, 306–309.

AZUMA A, YAMANO Y, YOSHIMURA A, HIBINO T, HISHIDA T, YAGITA H, OKUMARA K, SEYA T, KANNAGI R, SHIBUYA M AND KUDOH S. (1995). Augmented lung adenocarcinoma cytotoxicity by the combination of a genetically modified anti-Lewis Y antibody and antibodies to complement regulatory proteins. *Scand. J. Immunol.*, **42**, 202–208.

DALE TC, ROSEN JM, GUILLE MJ, LEWIN AR, PORTER AGC, KERR IM AND STARK GR. (1989). Overlapping sites for constitutive and induced DNA binding factors involved in interferon-stimulated transcription. *EMBO J.*, **8**, 831–839.

DEGOS L. (1990). Differentiating agents in the treatment of leukemia and myelodysplastic syndromes. *Leukemia Res.*, **14**, 731–733.

DIGNAM JD, LEBOVITZ RM AND ROEDER PG. (1983). Accurate transcription initiation by RNA polymerase II in a soluble extract from isolated mammalian nuclei. *Nucleic Acids Res.*, **11**, 1475–1489.

DONNADIEU N, PAESMANS M AND SOULIER JP. (1991). Chemotherapy of non-small lung cancer according to disease extent: a meta-analysis of the literature. *Lung Cancer*, **7**, 243–252.

EDELSON JD, SHANNON JM AND MASON RJ. (1988). Alkaline phosphatase: a marker of alveolar type II cell differentiation. *Am. Rev. Respir. Dis.*, **138**, 1268–1275.

GIARD DJ, AARONSON SA, TODARO GJ, ARNSTEIN P, KERSEY JH, DOSIK H AND PARKS WO. (1972). In vitro cultivation of human tumors: establishment of cell lines from a series of solid tumors. *J. Natl Cancer Inst.*, **51**, 1417–1423.

GINSBERG RJ, KRIS MG AND ARMSTRONG J. (1993). Cancer of the lung. In: *Cancer: Principles and Practice of Oncology*, 4th edn, Devota VT, Hellman S and Rosenberg SA. (eds), pp. 673–772, J.B. Lippincott: Philadelphia.

GOTO I, HOZUMI M AND HONMA Y. (1994). Selective effect of *O*-alkyl lysophospholipids on the growth of a human lung giant cell carcinoma cell line. *Anticancer Res.*, **14**, 357–362.

GUCHELAAR HJ, TIMMER-BOSSCHA H, DAM-MEIRING A, UGES DRA, OOSTERHUIS JW AND DEVRIES EGE. (1993). Enhancement of cisplatin and etoposide cytotoxicity after all-trans retinoic-acid-induced cellular differentiation of a murine embryonal carcinoma cell line. *Int. J. Cancer*, **55**, 442–447.

HANAI N, ISHIHARA T, UCHINO F, IMADA N, FUJIHARA S AND IKEGAMI J. (1979). Effects of dimethyl sulfoxide and colchicine on the resorption of experimental amyloid. Virchows Arch. *A Path. Anat. Histol.*, **384**, 45–52.

HIRAKI S, MIYAI M, SETO T, TAMURA T, WATANABE Y, OZAWA S, IKEDA H, NAKATA Y, OHNOSHI T AND KIMURA I. (1982). Establishment of human continuous cell lines from squamous cell, adeno- and small cell carcinoma of the lung and the results of hetero-transplantation. *Haigan*, **22**, 53–58.

HONMA Y, ONOZUKA Y, OKABE-KADO J, KASUKABE T AND HOZUMI M. (1991). Hemin enhances the sensitivity of erythroleukemia cells to 1-β-D-arabinofuranosylcytosine by both activation of deoxycytidine kinase and reduction of cytidine deaminase activity. *Cancer Res.*, **51**, 4535–4538.

HOZUMI M. (1983). Fundamentals of chemotherapy of myeloid leukemia by induction of leukemia cell differentiation. *Adv. Cancer Res.*, **38**, 121–169.

HUANG ME, YE Y, CHEN SR, CHAI JR, LU JX, ZHOA L, GU LJ AND WANG ZI. (1988). Use of all-trans-retinoic acid in the treatment of acute promyelocytic leukemia. *Blood*, **72**, 567–572.

ISOBE T AND OSSERMAN EF. (1976). Effects of dimethyl sulfoxide (DMSO) on Bence Jones protein, amyloid fibrils and casein-induced amyloidosis. In: *Amyloidosis*, O Wegelius, A Pasternach (eds) pp. 247–257, Academic Press: New York.

IWASAKI T, HAMANO T, AIZAWA K, KOBAYASHI K AND KAKISHITA E. (1994). A case of pulmonary amyloidosis associated with multiple myeloma successfully treated with dimethyl sulfoxide. *Acta Haematol.*, **91**, 91–94.

KASAHARA K, FUJIWARA Y AND NISHIO K. (1991). Metallothionein content correlates with the sensitivity of human small cell lung cancer cell lines to cisplatin. *Cancer Res.*, **51**, 3237–3242.

KESSLER DS, PINE R, PFEFFER LM, LEVY DE AND DARNELL JE. (1988). Cells resistant to interferon are defective in activation of a promoter-binding factor. *EMBO J.*, **7**, 3779–3783.

KLASTERSKY J, SCULIER JP, NICAISE C, WEERTS D, MAIRESSE M, LIBERT P, ROCMANS P, MICHEL J AND FERREMANS W. (1983). Combination chemotherapy with cisplatin etoposide and vindesine in non-small cell lung carcinoma. *Cancer Treat. Rep.*, **67**, 727–730.

KOYAMA H AND ONO T. (1976). Induction by short-chain fatty acids of alkaline phosphatase activity in cultured mammalian cells. *J. Cell. Phys.*, **88**, 49–56.

LEVY DE, KESSLER DS, PINE R AND DARNELL JE. (1988). Interferon-induced nuclear factors that bind a shared promoter element correlate with positive and negative transcriptional control. *Genes Dev.*, **2**, 383–393.

LEVY DE, KESSLER DS, PINE R AND DARNELL JE. (1989). Cytoplasmic activation of ISGF3, the positive regulator of interferon α-stimulated transcription, reconstituted in vitro. *Genes Dev.*, **3**, 1362–1371.

LIPPMAN SM, KAVANAGH JJ, PAREDES-ESPINOZA M, DELIGA-DILLO-MADRUENO F, PAREDES-CASILLAS P, HONG WK, HOLDENER E AND KRAKOFF IH. (1992*a*). 13-cis-Retinoic acid plus interferon alpha-2a: highly active systemic therapy for squamous cell carcinoma of the cervix. *J. Natl Cancer Inst.*, **84**, 241–245.

LIPPMAN SM, PARKINSON DR, ITRI LM, WEBER RS, SCHANTZ SP, OTA DM, SCHUSTERMAN MA, KRAKOFF IH, GUTTERMAN JU AND HONG WK. (1992*b*). 13-cis-Retinoic acid and interferon alpha-2a: effective combination therapy for advanced squamous cell carcinoma of the skin. *J. Natl Cancer Inst.*, **84**, 235–241.

LOETHER PJ AND EINHORN LH. (1984). Cisplatin. *Ann. Intern. Med.*, **100**, 704–713.

MCCORMICK C, FRESHNEY RI AND SPEIRS V. (1995). Activity of interferon α, interleukin 6 and insulin in the regulation of differentiation in A549 alveolar carcinoma cells. *Br. J. Cancer*, **1**, 232–239.

MARTYRE M-C AND WIETZERBIN J. (1994). Characterization of specific functional receptors for HuIFN-α on a human megakaryocytic cell line (Dami): expression related to differentiation. *Br. J. Haematol.*, **86**, 244–252.

OHNO R, YOSHIDA H, FUKUTANI H, NAOE T, OHSHIMA T, KYO T, ENDOH N, FUJIMOTO T, KOBAYASHI T, HIRAOKA A, MIZO-GUCHI H, KODERA Y, SUZUKI H, HIRANO M, AKIYAMA H, AOKI N, SHINDO H AND YOKOMAKU S. (1993). Multinstitutional study of all-trans retinoic acid as a differentiation therapy of refractory acute promyelocytic leukemia. *Leukemia*, **7**, 1772–1727.

OHTANI T, YAMADA R, TAIRA O, OOHMA M, TSUJI K AND HAYATA Y. (1976). Immune response in one case of advanced lung cancer. *Haigan*, **16**, 67–71.

OKABE-KADO J, HAYASHI M, HONMA Y AND HOZUMI M. (1986). Enhancement by hemin on the sensitivity of K562 human leukemic cells to 1-β-D-arabinofuranosylcytosine. *Cancer Res.*, **46**, 1239–1243.

OKABE-KADO J, HAYASHI M, HONMA Y, HOZUMI M AND TSURUO T. (1991). Inhibition by erythroid differentiation factor (activin A) of p-glycoprotein expression in multidrug-resistant human K562 erythroleukemia cells. *Cancer Res.*, **51**, 2582–2586.

REUBEN RC, WIFE RL, BRESLOW R, RIFKIND RA AND MARKS PA. (1976). A new group of protein inducers of differentiation in murine erythroleukemia cells. *Proc. Natl Acad. Sci. USA*, **73**, 862–866.

RUTHERFORD MN, HANNIGAN GE AND WILLIAMS BRG. (1988). Interferon-induced binding of nuclear factors to promoter elements of the 2-5A synthetase gene. *EMBO J.*, **7**, 751–759.

SAKIYAMA S, NAKAMURA Y AND YASUDA S. (1986). Expression of epidermal growth factor receptor gene in cultured human lung cancer cells. *Jpn. J. Cancer Res.*, **77**, 965–969.

SAMID D, SHACK S AND SHERMAN LT. (1992). Phenylacetate: a novel nontoxic inducer of tumor cell differentiation. *Cancer Res.*, **52**, 1988–1992.

TERASAKI T, SHIMOSATO Y, NAKAJIMA T, TSUMURAYA M, MORINAGA S, HIROHASHI S, YAMAGOCHI K, KATO K, ICHINOSE H AND NAGATSU T. (1986). Changes in cell characteristics due to culture conditions in cell lines from human small cell lung cancer. *Jpn. J. Clin. Oncol.*, **16**, 203–212.

WORRALL PM. (1982). Single-dose ifosfamide: efficacy studies in non small lung cancer. *Semin. Oncol.*, **9** (Suppl. 1), 56–60.

XU B, GRANDER D, SANGFELT O AND EINHORN S. (1994). Primary leukemia cells resistant to α-interferon in vitro are defective in the activation of the DNA-binding factor interferon-stimulated gene factor 3. *Blood*, **84**, 1942–1949.

YAMADA T, HIROHASHI S AND SHIMOSATO Y. (1985). Giant carcinomas of the lung producing colony-stimulating factor *in vitro* and *in vivo*. *Jpn. J. Cancer Res.*, **76**, 967–976.

YOSHIOKA S. (1989). Studies on thiol protease inhibitor isolated from human lung cancer cell lines. *Hiroshima J. Med. Sci.*, **37**, 199–215.

YOUNG CW, FANUCCHI MP, WALSH D, BALTZER L, YALDAEI S, STEVENS Y-W, GORDON C, TONG W, RIFKIND RA AND MARKS PA. (1988). Phase I trial and clinical pharmacological evaluation of hexamethylene bisacetamide administration by ten-day countinous intravenous infusion at twenty-eight-day intervals. *Cancer Res.*, **8**, 7304–7309.